# Promotion of the lipase-catalyzed hydrolysis of conjugated linoleic acid l-menthyl ester by addition of an organic solvent

**DOI:** 10.1186/2193-1801-1-67

**Published:** 2012-12-14

**Authors:** Takashi Kobayashi, Toshihiro Nagao, Yomi Watanabe, Yuji Shimada

**Affiliations:** 1 Division of Food Science and Biotechnology, Graduate School of Agriculture, Kyoto University, Sakyo-ku, Kyoto, 606-8502 Japan; 2 Osaka Municipal Technical Research Institute, 1-6-50 Morinomiya, Joto-ku, Osaka, 536-8553 Japan; 3 Okamura Oil Mill Co., Ltd, 4-5, Kawaharacho, Kashiwara, Osaka, 582-0004 Japan

**Keywords:** Conjugated linoleic acid, Ester, Hydrolysis, Lipase, Organic solvent

## Abstract

Conjugated linoleic acid l-menthyl ester was hydrolyzed in water by the lipase from *Candida rugosa* with the addition of an organic solvent. The degree of hydrolysis (yield) greatly improved when a tertiary alcohol, such as *t*-butyl alcohol, was added. However, the addition of a less polar solvent, such as hexane, decreased the degree of hydrolysis, and some water-miscible solvents, such as acetone, caused inactivation of the lipase. With the addition of *t*-butyl alcohol, the reaction mixture formed a one- or two-phase system, and the mixing ratio of substrates and *t*-butyl alcohol determined the number of phases. Although the degree of hydrolysis at 10 d was higher in the one-phase system, the initial reaction rate was generally lower. Meanwhile, the reaction was much faster in the two-phase system while maintaining a moderate degree of hydrolysis.

## Background

Commercially available conjugated linoleic acid (CLA) contains two structural isomers (9-*cis*,11-*trans*- and 10*t*,12*c*-CLA). It has been reported that each isomer has different physiological activities, such as an anti-cancer activity (Soel et al. 2007), decreasing the body fat content (Park et al. 1999), and suppressing the development of hypertension (Nagao et al. 2003a). To develop nutraceuticals containing the two CLA isomers of arbitrary amounts, a fractionation process of the CLA isomers is needed.

Some fractionation processes have been developed using the lipase-catalyzed selective esterification (Haas et al. 1999; Kobayashi et al. 2006; Nagao et al. 2003b). In these processes, lipases, such as from *Candida rugosa*, one of the most effective lipases for the esterification of CLA, were used, and some alcohols were used for the hydroxyl donors. l-Menthol is one these alcohols and very effective for the large-scale fractionation by selective esterification with CLA (Kobayashi et al. 2006). In this process, the alkali hydrolysis of CLA menthyl ester was needed to recover CLA as a free fatty acid. However, the hydrolysis requires heating under a strong alkaline condition, which may cause decomposition or isomerization of CLA. The lipase-catalyzed hydrolysis of CLA menthyl ester is considered to avoid these drawbacks. However, a degree of hydrolysis (yield) for CLA menthyl ester would be low in an oil/water two-phase system because the degree of the reverse reaction (esterification) is very high (*ca*. 80%) at equilibrium (Kobayashi et al. 2007).

In general, the hydrolytic reaction system by lipases consists of two phases, i.e., water and oil phases in a neat reaction mixture. The water content in the oil phase is fixed in this case. However, addition of an organic solvent would promote the partition of water from the water phase to the oil phase, which results in changing the water content in the oil phase. To promote the hydrolysis, this change would be effective because the water content in the oil phase plays an important role in determining the degree of reaction in the two-phase system (Kobayashi et al. 2007). In this study, some organic solvents were added to the reaction system at various mixing ratios of the substrates and the solvent in order to evaluate the performance of the lipase-catalyzed hydrolysis of CLA menthyl ester.

## Materials and methods

### Materials

CLA in a free fatty acid form was a gift from The Nisshin OilliO Group, Ltd. (Tokyo, Japan), the fatty acid contents of which were 33% 9*c*,11*t*-CLA, 34% 10*t*,12*c*-CLA, 4.9% other CLAs, 16% oleic acid, 6.4% palmitic acid, 2.7% stearic acid, and 2.2% other fatty acids. Lipase from *Candida rugosa* (Lipase-OF) was from Meito Sangyo (Aichi, Japan).

### Preparation of CLA menthyl ester

Lipase from *C*. *rugosa* (1.8×10^4^ U/g-powdery enzyme) was used as an aqueous solution (1.8×10^4^ U/mL) in which 1 U lipase was defined as the amount of lipase which liberated 1 μmol free fatty acid/min during the hydrolysis of olive oil. Esterification of CLA was performed by stirring 100 g (0.36 mol) CLA, 55.8 g (0.36 mol) l-menthol, and 8.67 mL lipase solution at 500 rpm and 30°C for 7 d under a nitrogen atmosphere. After the reaction, the reaction mixture was separated into the oil and water phases by centrifugation. CLA menthyl ester and unreacted l-menthol were extracted with hexane after adding a 0.1 M sodium hydroxide aqueous solution to the oil phase to remove any unreacted CLA (free fatty acid). The mixture of CLA menthyl ester and l-menthol was distilled under vacuum at 150°C and 500 Pa to obtain CLA menthyl ester as a distillation residue.

### Hydrolysis of CLA menthyl ester in the presence of an organic solvent

CLA menthyl ester, organic solvent, and water at specific amounts were weighed into a screw-capped vial. In a typical procedure, 2 g CLA menthyl ester, 2 g *t*-butyl alcohol and 1 g water were used. To the substrate mixture was added the powdery lipase from *C*. *rugosa* at 8.9×10^3^ U/g-ester. The mixture was then stirred at 500 rpm and 30°C for 10 d under a nitrogen atmosphere. The organic solvents used in this reaction were *t*-butyl alcohol, *t*-amyl alcohol, diacetone alcohol, diethyl ether, diisopropyl ether, hexane, cyclohexane, acetone, 2-butanone, cyclohexanone, and acetonitrile. The reaction without any organic solvent was also tested as the control. At appropriate intervals, the reaction mixture (*ca*. 250 mg) was sampled and analyzed to estimate the initial reaction rate, water content in the oil phase, and degree of hydrolysis.

### Analysis

The water content in the oil phase of the reaction mixture was measured by Karl-Fischer titration using a CA-07 moisture meter (Mitsubishi-Kagaku, Tokyo). The measurement was performed at least five times using an *ca*. 40 mg oil phase in each measurement, and the median value was adopted as the water content.

The degree of hydrolysis was determined by gas chromatography (GC). Prior to applying a sample to the GC, the reaction mixture was diluted 10 times with hexane. The diluted sample was centrifuged at 9000×*g* for 2 min to separate it into the hexane and water phases. After removing the water phase, solvents in the hexane phase were removed under vacuum. Free fatty acids in the residual oil underwent methylation as follows (Kobayashi et al. 2011): Ten milligrams of the oil was dissolved in 1 mL toluene/methanol (3:2, v/v). The solution was mixed well with a 25 μL (trimethylsilyl)-diazomethane diethyl ether solution (2 M), and the mixture was stored for 10 min at room temperature. After methylation, the mixture was applied to a 6890N GC (Agilent Technologies, CA, USA) connected to a DB-23 column (0.25 mm I.D. × 30 m; Agilent). The column temperature was raised as follows: 150 to 200°C, 10°C/min; 200 to 210°C, 2°C/min; 210 to 230°C, 15°C/min; kept at 230°C for 10 min.

## Results and discussion

### Effect of the type of organic solvent on the hydrolysis

Various kinds of organic solvents including hydrocarbons, ketones, tertiary alcohols, acetonitrile, and ethers were tested in order to evaluate the solvent effect on the hydrolysis of CLA menthyl ester. The reaction mixture consisted of two phases (oil/water) during the reaction for all cases when the reaction was performed using 2 g CLA menthyl ester, 2 g organic solvent, 1 g water, and 1.8×10^4^ U lipase. Table 1 shows the degree of hydrolysis at 1 d. Organic solvents having a tertiary hydroxyl group (*t*-butyl alcohol, *t*-amyl alcohol, and diacetone alcohol) greatly promoted the hydrolysis. Especially, when *t*-butyl alcohol or *t*-amyl alcohol, which was one of the water-miscible solvents, was used, the degree of hydrolysis at 1 d reached *ca*. 30%. Meanwhile, when a relatively less polar solvent, such as diisopropyl ether, hydrocarbons, or cyclohexanone, was used, the degree of hydrolysis was lower than that of the control (<10%). One of the reasons may be as follows: The lipase-catalyzed hydrolysis occurs at the interface of the oil/water phases, and the reaction behavior is greatly affected by the water content in the oil phase (Kobayashi et al. 2007). Although water activity is the most appropriate for evaluating the degree of hydrolysis in these systems, it is very difficult to determine all the reactant activities, including water activity, in a multi-component system. Therefore, we used the water content in the following study. Because many of these less polar solvents are only slightly miscible with water, these solvents lower the water content in the oil phase, resulting in the lower degree of hydrolysis. When diethyl ether, acetone, 2-butanone, and acetonitrile, which are water-miscible or miscible with water to a certain degree, were used, the hydrolysis only slightly proceeded. The low degree of hydrolysis may be due to the inactivation of the lipase by an organic solvent.

**Table 1:** **Effect of the type of an organic solvent on the degree of hydrolysis at 1 d**

Solvent	Degree of hydrolysis(%)
Control	14.0±1.5
*t*-Butyl alcohol	32.2±2.0
*t*-Amyl alcohol	31.5±3.0
Diacetone alcohol	20.5±2.5
Hexane	9.2±2.2
Cyclohexane	6.3±1.1
Cyclohexanone	6.1±1.5
Acetone	0.6±0.1
2-Butanone	1.0±0.2
Diisopropyl ether	7.5±1.0
Diethyl ether	0.7±0.1
Acetonitrile	0.7±0.2

The reaction was performed in triplicate by mixing 2 g CLA menthyl ester, 2 g organic solvent, 1 g water, and 1.8×10^4^ U lipase at 30°C for 1 d.

These results indicate that lipase from *C*. *rugosa* is not completely inactivated in a tertiary alcohol. On the other hand, other water miscible organic solvents, such as acetone and acetonitrile, readily inactivate the lipase. These facts contrast the fact that lipase from *Candida antarctica* (fraction B) shows a high catalytic activity not only in *t*-butyl alcohol, but also in acetone and acetonitrile (Kobayashi et al. 2010; Zhu et al. 2012). Based on these results, *t*-butyl alcohol was used for the following studies.

### State of the reaction mixture

Because *t*-butyl alcohol is a water-miscible solvent and also miscible with CLA menthyl ester, the addition of *t*-butyl alcohol greatly changes the state of the reaction mixture which consists of two phases, i.e., aqueous and oil phases. When a small amount of *t*-butyl alcohol is added to the mixture, water and CLA menthyl ester are partitioned into the oil and aqueous phases, respectively. Meanwhile, the mixture will become one phase when more *t*-butyl alcohol is added. Therefore, the initial ratios of *t*-butyl alcohol, CLA menthyl ester and water were changed, and effects of the ratio on the hydrolysis were discussed from the viewpoint of the state of the reaction mixture, the initial reaction rate, and the degree of hydrolysis.

**Figure 1:**
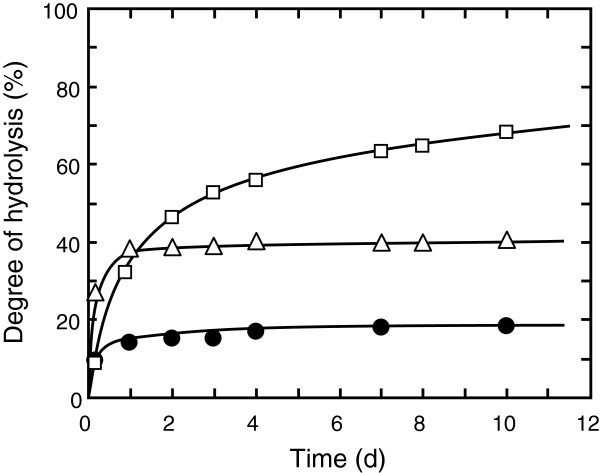
**Time courses for the hydrolysis of CLA menthyl ester in the presence of*t*-butyl alcohol.** The reaction was performed at 30°C by mixing CLA menthyl ester, water, *t*-butyl alcohol, and 8.9×10^3^ U-lipase/g-ester. Symbols *black circle*, △, and □ represent the results obtained using the mixtures of CLA menthyl ester : H_2_O : *t*-butyl alcohol at the initial weight ratios of 2 : 1 : 0 (control), 2 : 1 : 1, and 2 : 1 : 15, respectively.

Hydrolysis was performed by changing the mixing ratio of CLA menthyl ester (1–2 g), *t*-butyl alcohol (0–30 g), and water (0.5–10 g). Typical time courses are shown in Figure 1. The reaction rate and degree (yield) of hydrolysis were greatly influenced by the addition of *t*-butyl alcohol. When a relatively small amount of *t*-butyl alcohol (25 wt%) was added, the number of phases was two. The reaction in this composition proceeded very fast and reached equilibrium within 1 d. On the other hand, when a large amount of *t*-butyl alcohol (83 wt%) was added, the reaction mixture became one phase. The hydrolysis slowly proceeded and did not reach equilibrium within 10 d. Although the reaction was slow, the degree of hydrolysis continued to increase during the reaction and reached 68% at 10 d. These results indicate that the lipase from *C*. *rugosa* does not completely inactivate even at a high concentration of *t*-butyl alcohol, and that *t*-butyl alcohol positively affects the promotion of the hydrolysis. To shorten the reaction time, addition of much enzyme would be effective. Meanwhile, the control reaction almost reached equilibrium within 4 d, and the degree of hydrolysis was lower (18% at 10 d) than that in the presence of *t*-butyl alcohol.

**Figure 2:**
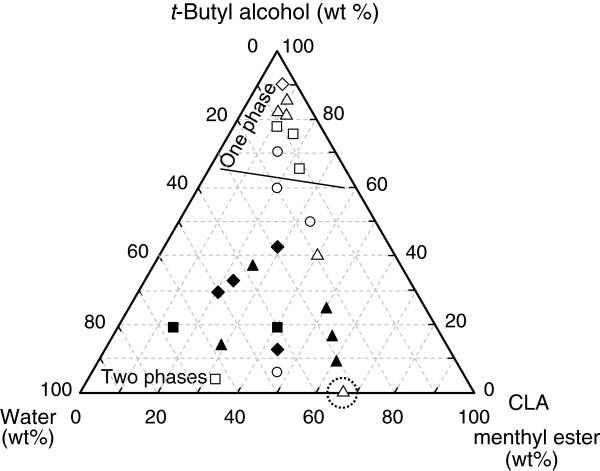
**Dependence of the initial reaction rate on the mixing ratio of substrates and*t*-butyl alcohol.** The initial rate without *t*-butyl alcohol (control) was 9.68 nmol h^-1^ U^-1^), and its relative rate was regarded as 1.0. Symbols *white diamond*, △, □, ○, *black diamond*, *black triangle*, and *black square* represent the relative initial rate in the ranges of 0.30–0.99, 1.0–1.9, 2.0–2.9, 3.0–5.0, 7.0–7.9, 8.0–8.9, and 10–11, respectively. The symbol inside the dotted circle represents the control. The reaction condition was the same as that in Fig. 1 except for the mixing ratio.

**Figure 3:**
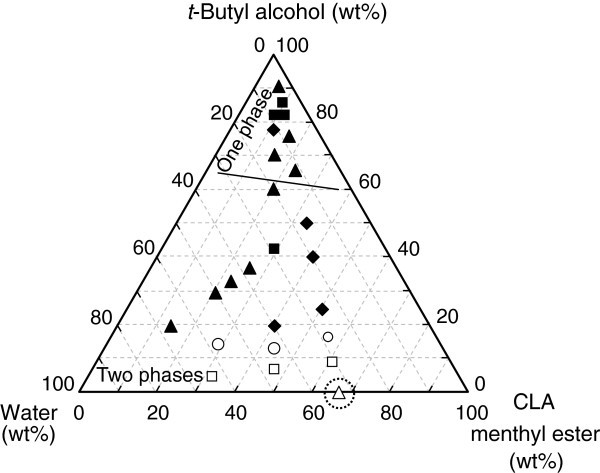
**Dependence of the degree of hydrolysis on the mixing ratio of substrates and*t*-butyl alcohol.** Symbols △, □, ○, *black diamond*, *black triangle*, and *black square* represent the degree of hydrolysis at 10 d in the ranges of 10–19%, 20–29%, 30–39%, 40–49%, 50–59%, and 60–70%, respectively. The symbol inside the dotted circle represents the control. The reaction condition was the same as that in Figure 1 except for the mixing ratio.

Figures 2 and 3 show ternary diagrams for the relative initial reaction rate and degree of hydrolysis at 10 d, respectively, for various mixing ratios of CLA menthyl ester, water, and *t*-butyl alcohol. The mixing ratio greatly affected the initial reaction rate and the degree of hydrolysis in addition to the number of phases in the reaction mixture (one or two phases). When the content of *t*-butyl alcohol was less than *ca*. 60 wt%, the reaction mixture consisted of two phases, but when a large amount of *t*-butyl alcohol was added, it became one phase. The effect of *t*-butyl alcohol is separately discussed for the one- and two-phase cases in the following sections.

### Hydrolysis in one-phase system

In the one-phase system, the initial reaction rates were *ca*. 1–4 fold higher than that of the control in most cases (Figure 2). However, the significant addition of *t*-butyl alcohol tended to decrease the initial rate. Meanwhile, the degree of hydrolysis at 10 d greatly improved to 42–68% in all cases compared to that of the control as shown in Figure 3, maybe due to the high concentration of water in the reaction mixture. However, the addition of a large amount of *t*-butyl alcohol lowers the substrate concentration. Therefore, performing the reaction under the one-phase state is not practical.

There are some possibilities for the low reaction rate at the higher ratio of *t*-butyl alcohol: 1) Low substrate concentration is directly related to the low reaction rate. 2) Water-miscible organic solvents cause unfolding of the protein (Klyosov et al. 1975) or elimination of water molecules from an enzyme molecule that are essential to maintain its catalytic activity (Krishna et al. 2001). Therefore, lipase may be gradually inactivated by the significant addition of *t*-butyl alcohol. 3) A lipase molecule is generally adsorbed on the interface between the oil/water phases, and the interfacial activation produces the catalytic activity (Grochulski et al. 1993). In the one-phase system, however, there is no interface, and the behavior of the interfacial activation would be different from that in the typical two-phase system.

### Hydrolysis in two-phase system

The initial reaction rates in the two-phase system were much improved for all cases compared to that of the control (Figure 2). Especially when the ratio of *t*-butyl alcohol was 9–50 wt%, the reaction rates tended to greatly increase, and the maximum reaction rate reached *ca*. 10.8 times that of the control. These results would support the fact that the addition of a small amount of *t*-butyl alcohol activates the lipase from *C*. *rugosa*, and that the formation of the interface between the two phases is very important for the lipase to sufficiently exhibit its catalytic activity even in the presence of *t*-butyl alcohol.

**Figure 4:**
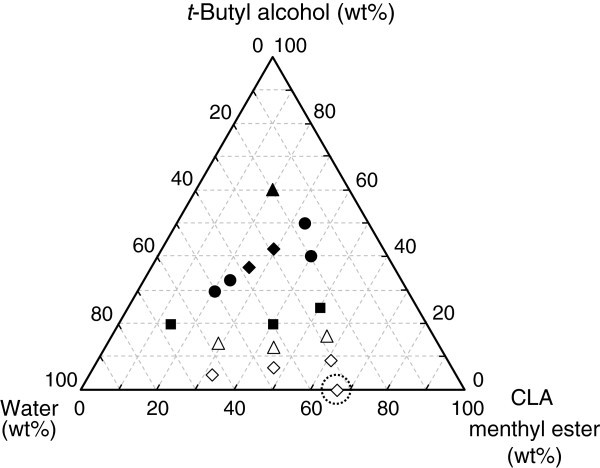
**Initial water contents in the oil phase in a two-phase system at 30°C.** Symbols *white diamond*, △, *black square*, *black circle*, *black diamond*, and *black triangle* represent the water content in the ranges of 0.20–0.99 wt%, 1.0–1.9 wt%, 2.0–4.9 wt%, 5.0–9.9 wt%, 10–14.9 wt%, and 15–20 wt%, respectively. The symbol inside the dotted circle represents the control.

The degree of hydrolysis is next discussed. The degrees of hydrolysis at 10 d were 24–61% in the presence of *t*-butyl alcohol and were higher than that of the control (Figure 3). In the two-phase system, the partition of water between the oil/water phases is an important factor affecting the reaction behavior. Water that is supplied to a catalytic center of a lipase molecule is transported through the oil phase (Kobayashi et al. 2007). Therefore, it is favorable to increase the water content in the oil phase for improving the hydrolysis in the two-phase system. The initial water content was 0.2 wt% when *t*-butyl alcohol was not added, while it became 0.3–27 wt% with the addition of *t*-butyl alcohol (Figure 4). When the water content was greater than 2 wt%, the degree of hydrolysis reached 40–61%. In these cases, an unstable emulsion was formed, indicating that the formation of an emulsion would be an effective way to improve both the reaction rate and degree of hydrolysis. Based on these results, increasing the water content can be achieved by the addition of *t*-butyl alcohol to a certain amount, and its effectiveness has been revealed for significantly improving the hydrolysis of CLA menthyl ester. The same procedure may contribute to hydrolyze other esters in order to recover a free fatty acid and/or an alcohol.

## Conclusion

In conclusion, the addition of an organic solvent greatly affected the degree of hydrolysis of CLA menthyl ester by the lipase from *C*. *rugosa*. When tertiary alcohols, such as *t*-butyl alcohol, were used, the degree of hydrolysis improved. The mixing ratio of substrates and *t*-butyl alcohol affected the number of phases, and the state of the phase much influenced the initial reaction rate and degree of hydrolysis. The initial reaction rate was generally lower in the one-phase system. In the two-phase system, the reaction was much faster, and a higher degree of hydrolysis could be achieved when the reaction mixture formed an emulsion. Therefore, to efficiently perform the hydrolysis, it is favorable to adopt the two-phase system in an emulsified form.
